# Supplementary material for: Co-ordinated control of the ADP-heptose/ALPK1 signalling network by the E3 ligases TRAF6, TRAF2/c-IAP1 and LUBAC
Source: Biochem J. 2022 Oct 21;479(20):2195–216. doi: 10.1042/BCJ20220401 (PMC9704527; doi:10.1042/BCJ20220401)
Supplement: Supplementary Material [file BCJ-479-2195-s1.pdf]

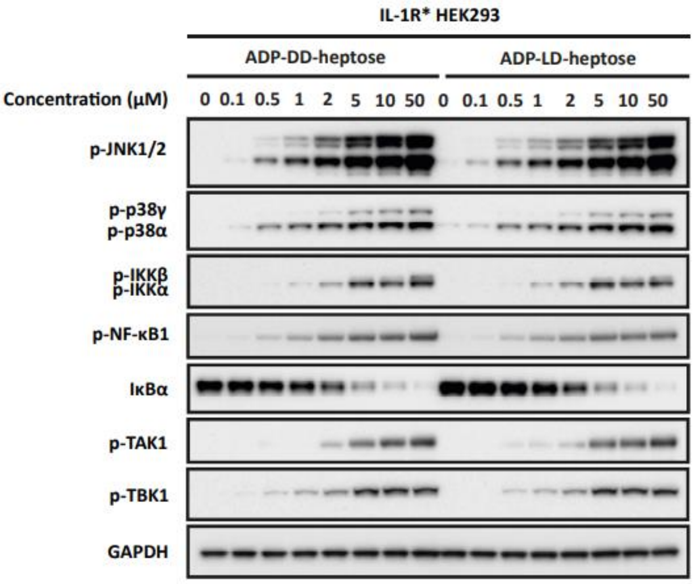

Figure S1. Concentration of ADP-DD-heptose and ADP-LD-heptose required for maximal activation of MAP kinases and the canonical IKK complex. IL-1R* HEK293 cells were stimulated for 30 min with the indicated concentrations of ADP-DD-heptose or ADP-LD-heptose. Cell extracts were analysed by SDS-PAGE and immunoblotted with antibodies recognising the proteins indicated. The molecular mass markers needed to gauge the size of each band are shown in Fig S7.

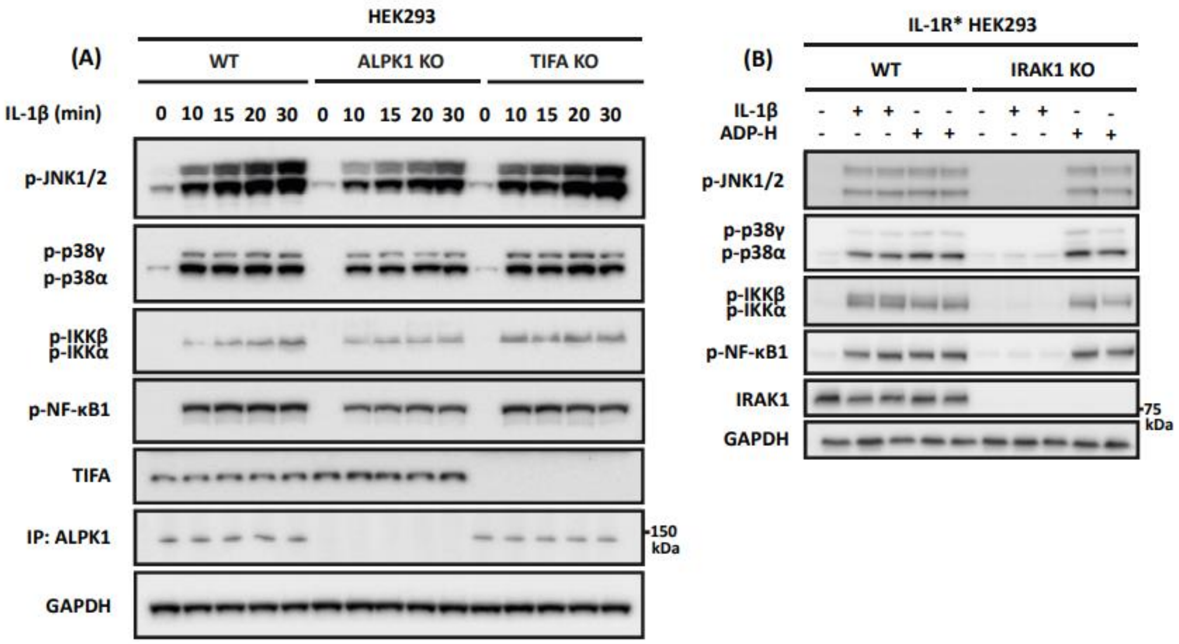

Figure S2. ALPK1 and TIFA are not required for IL-1β signalling and IRAK1 is not required for ADP-heptose signalling. (A) Parental, ALPK1 KO and TIFA KO HEK293 cells were stimulated with IL-1β the times indicated. Cell extracts were then analysed by SDS-PAGE and immunoblotting with the antibodies indicated. ALPK1 was immunoblotted after first immunoprecipitating it from the cell extracts. (B) As in A, except that parental and IRAK1 KO IL-1R* HEK293 cells were stimulated for 20 min with ADP-H or IL-1β. The molecular mass markers needed to gauge the size of each band are shown in Fig S7.

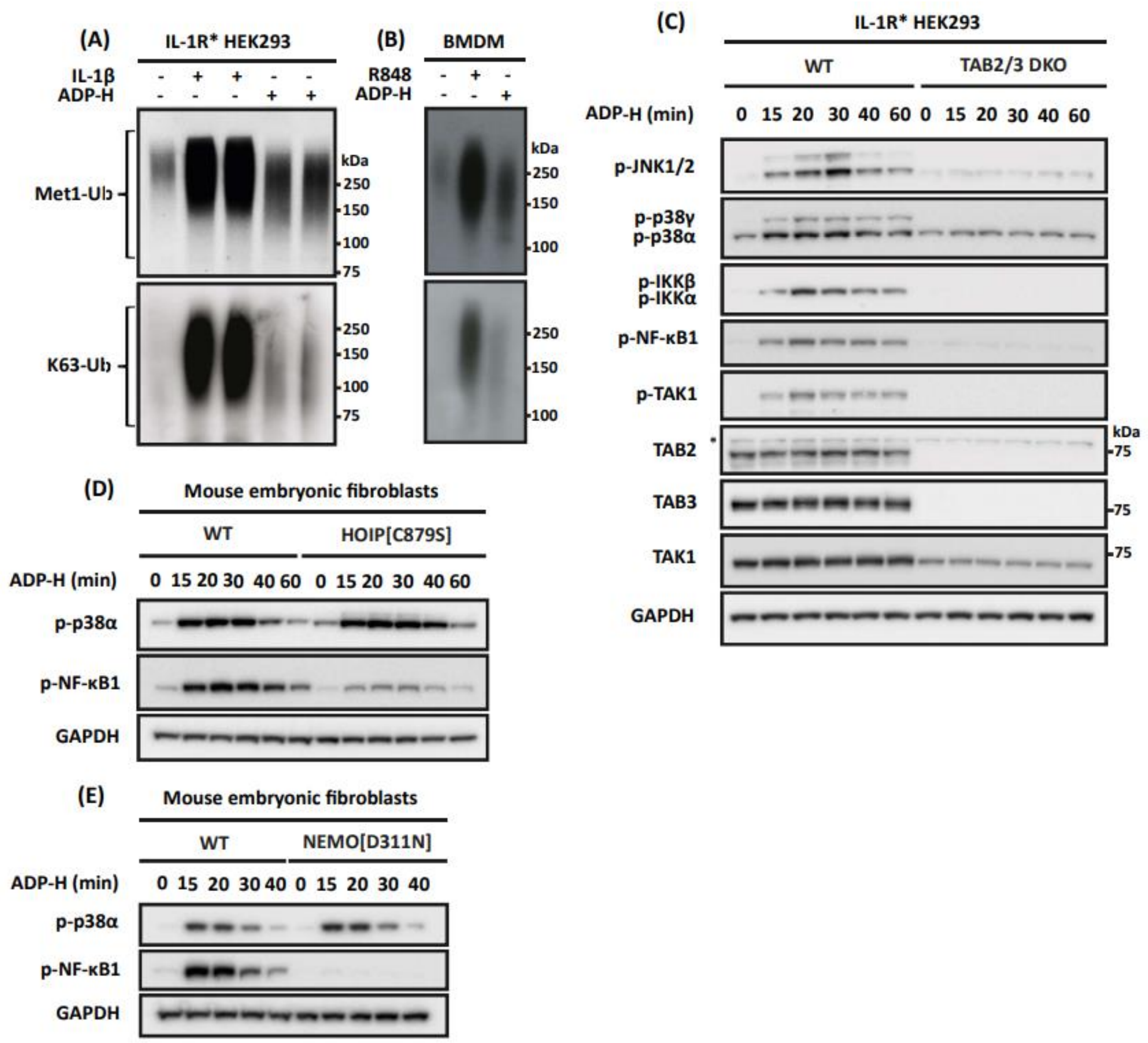

Figure S3. ADP-heptose signalling induces the formation of Lys63-linked and Met1-linked ubiquitin chains and requires the expression of TAB2 and TAB3, the E3 ligase activity of HOIP and the interaction of ubiquitin chains with NEMO. **(A)** IL-1R* HEK293 cells were stimulated for 15 min with ADP-heptose (ADP-H) or IL-1β. Ubiquitin chains were captured from the cell extracts on Halo-NEMO beads (see methods) and detected by SDS-PAGE and immunoblotting with antibodies recognising K63- or M1-linked ubiquitin oligomers. **(B)** As in A, but using primary BMDM from WT mice stimulated for 15 min with ADP-H or R848. (**C**) Parental and TAB2/3 double KO IL-1R* HEK293 cells were stimulated with ADP-H for the times indicated and immunoblotting of the cell extracts performed with the antibodies indicated. An asterisk indicates protein(s) recognised non-specifically by an antibody. **(D, E)** MEFs from WT and HOIP[C879S] (**D**) or WT and NEMO[D311N] **(E)** mice were stimulated for the times indicated with ADP-H and immunoblotting of the cell extracts performed with the antibodies indicated. The molecular mass markers needed to gauge the size of each band are shown in Fig S7.

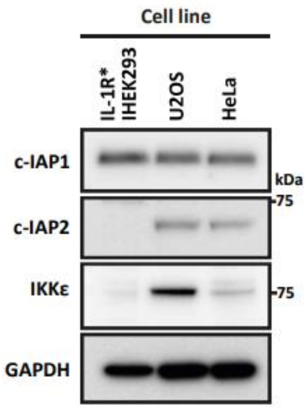

Figure S4. Relative levels of expression of c-IAP1, c-IAP2 and IKKε in three human cell lines. Cell extracts from three human cell lines were analysed by SDS-PAGE and immunoblotted with antibodies recognising the proteins indicated. The molecular mass markers needed to gauge the size of each band are shown in Fig S7.

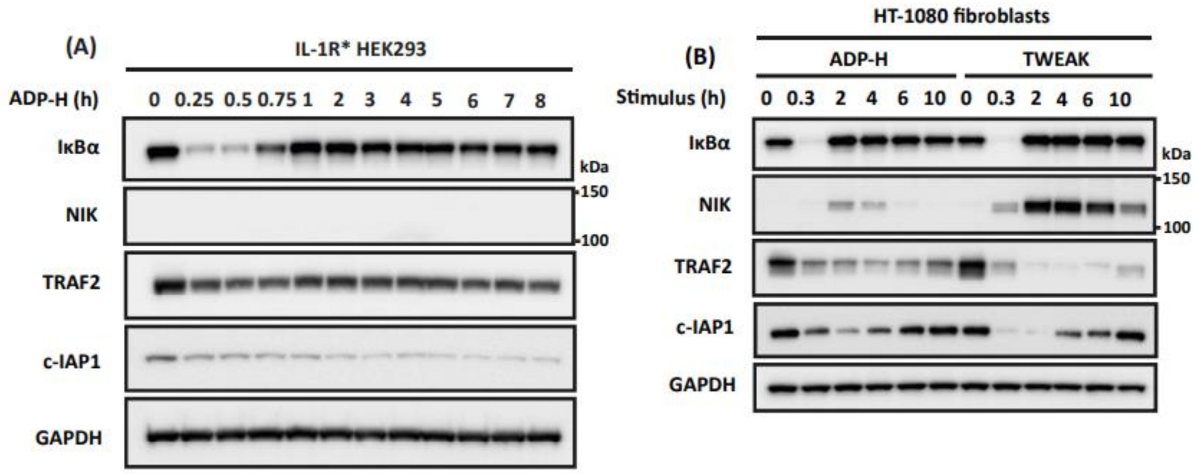

Figure S5. ADP-heptose does not activate the non-canonical NF-κB signalling pathway in HEK293 cells but activates it weakly in HT-1080 fibroblasts. (A) IL-1R* HEK293 cells were stimulated for the times indicated times with ADP-heptose (ADP-H) and the cell extracts analysed by SDS-PAGE and immunoblotting with antibodies recognising the proteins indicated. **(B)** As in A except that HT-1080 cells were stimulated with ADP-H or TWEAK.

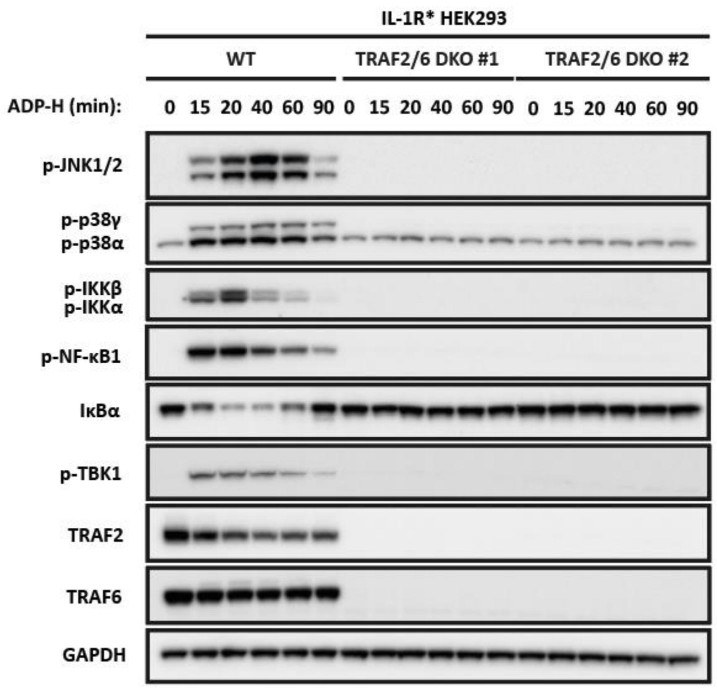

Figure S6. **ADP-heptose signalling is disrupted in cells lacking TRAF2 and TRAF6.** Parental and two different clones of TRAF2/TRAF6 double KO IL-1R* HEK293 cells were stimulated with ADP-H for the times indicated. The cell extracts were subjected SDS-PAGE and immunoblotting with the antibodies indicated. ADP-H did not increase the basal level of TBK1 phosphorylation significantly in the TRAF2/TRAF6 double knockout cells.

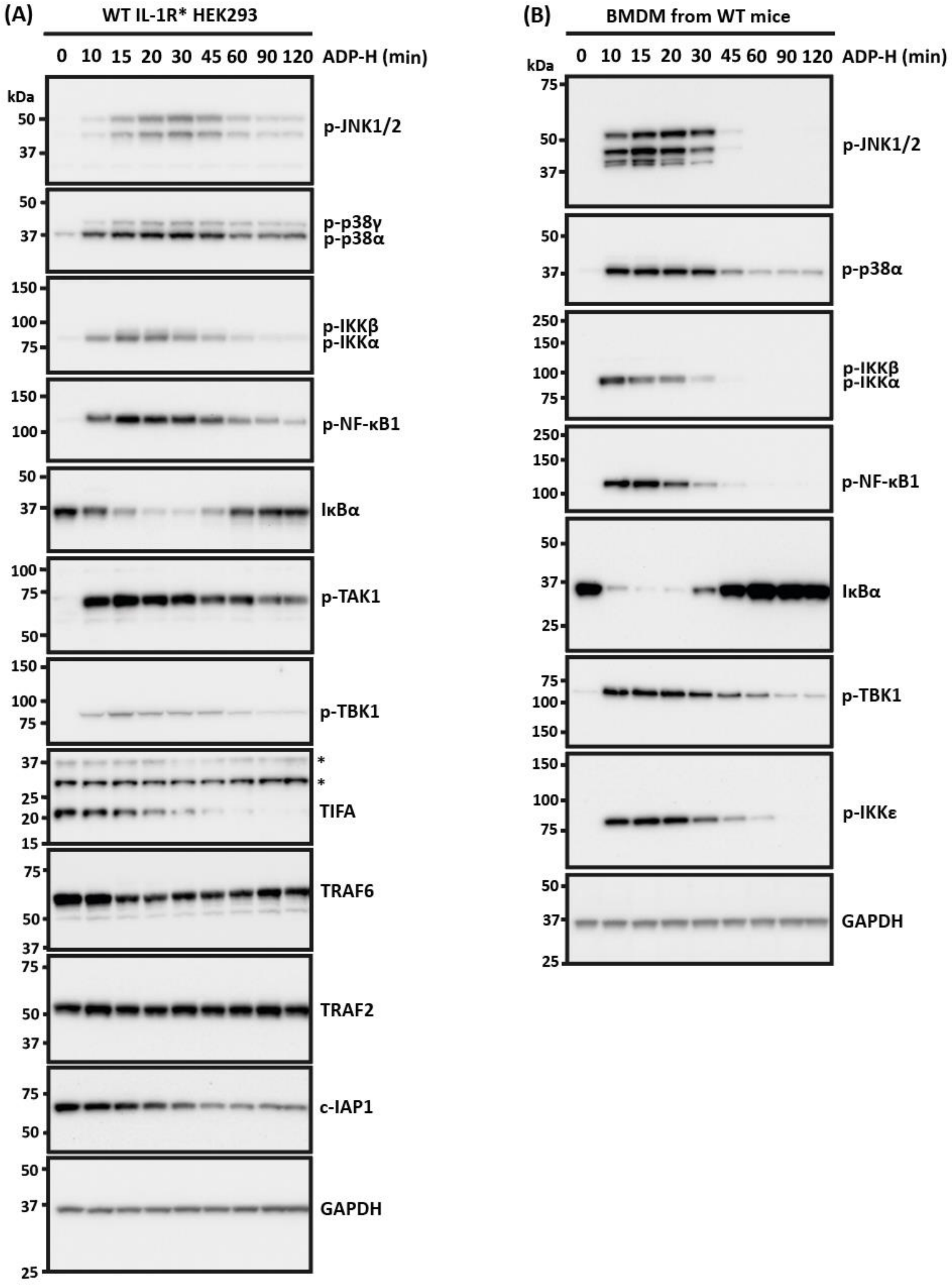

Figure S7. **Immunoblotting of the proteins used to monitor activation of the ADP-heptose signalling pathway in this paper.** IL-1R* HEK293 cells **(A)** or mouse BMDM **(B)** were stimulated for the indicated times with ADP-H and the cell extracts analysed by SDS-PAGE and immunoblotting with antibodies recognising the proteins indicated. Molecular mass markers are included to gauge the sizes of each protein. Some of these immunoblots are also presented in Fig 1A and 1B.
